# Human metapneumovirus M2-2 protein inhibits RIG-I signaling by preventing TRIM25-mediated RIG-I ubiquitination

**DOI:** 10.3389/fimmu.2022.970750

**Published:** 2022-08-15

**Authors:** Yukie Tanaka, Naoko Morita, Yoshinori Kitagawa, Bin Gotoh, Takayuki Komatsu

**Affiliations:** ^1^ Department of Integrative Vascular Biology, Faculty of Medical Sciences, University of Fukui, Fukui, Japan; ^2^ Department of Microbiology and Immunology, Aichi Medical University School of Medicine, Aichi, Japan; ^3^ Division of Microbiology and Infectious Diseases, Department of Pathology, Shiga University of Medical Science, Shiga, Japan

**Keywords:** M2-2 protein, rig-i, trim25, mavs, interferon, human metapneumovirus, human respiratory syncytial virus, paramyxoviridae

## Abstract

Retinoic acid-inducible gene I (RIG-I) is a receptor that senses viral RNA and interacts with mitochondrial antiviral signaling (MAVS) protein, leading to the production of type I interferons and inflammatory cytokines to establish an antiviral state. This signaling axis is initiated by the K63-linked RIG-I ubiquitination, mediated by E3 ubiquitin ligases such as TRIM25. However, many viruses, including several members of the family *Paramyxoviridae* and human respiratory syncytial virus (HRSV), a member of the family *Pneumoviridae*, escape the immune system by targeting RIG-I/TRIM25 signaling. In this study, we screened human metapneumovirus (HMPV) open reading frames (ORFs) for their ability to block RIG-I signaling reconstituted in HEK293T cells by transfection with TRIM25 and RIG-I CARD (an N-terminal CARD domain that is constitutively active in RIG-I signaling). HMPV M2-2 was the most potent inhibitor of RIG-I/TRIM25-mediated interferon (IFN)-β activation. M2-2 silencing induced the activation of transcription factors (IRF and NF-kB) downstream of RIG-I signaling in A549 cells. Moreover, M2-2 inhibited RIG-I ubiquitination and CARD-dependent interactions with MAVS. Immunoprecipitation revealed that M2-2 forms a stable complex with RIG-I CARD/TRIM25 *via* direct interaction with the SPRY domain of TRIM25. Similarly, HRSV NS1 also formed a stable complex with RIG-I CARD/TRIM25 and inhibited RIG-I ubiquitination. Notably, the inhibitory actions of HMPV M2-2 and HRSV NS1 are similar to those of V proteins of several members of the *Paramyxoviridae* family. In this study, we have identified a novel mechanism of immune escape by HMPV, similar to that of *Pneumoviridae* and *Paramyxoviridae* family members.

## Introduction

Human metapneumovirus (HMPV) is a leading cause of upper and lower respiratory tract infections in humans, particularly in infants, young children, the elderly, and immunocompromised individuals. HMPV belongs to the family *Pneumoviridae* in the order *Mononegavirales* and was first isolated in 2001 from children with respiratory ailments (1). This virus is the second most detected human respiratory pathogen after human respiratory syncytial virus (HRSV)—a member of the same family—and is responsible for a significant percentage of bronchiolitis-related hospitalizations in infants and young children (2–4). Additionally, HMPV induces poor innate immune responses, thereby affecting adaptive immunity and interferon (IFN) production (5). However, despite the similarities between HMPV and several other viruses, such as HRSV and parainfluenza viruses—members of the family *Paramyxoviridae*, in the same order—the mechanisms by which HMPV evades the host immune system remain largely unclear.

Upon detecting viral RNA in the cytoplasm, retinoic acid-inducible gene I (RIG-I)-like receptors (RLRs), including RIG-I and melanoma differentiation-associated gene 5 (MDA5), establish an antiviral state by triggering signaling cascades that induce the expression of type I IFNs and inflammatory cytokines (6–9). Members of the families *Paramyxoviridae* and *Pneumoviridae* harbor a 5′-triphosphate moiety, which is a non-self-RNA signature specific for RIG-I; therefore, they are speculated to be recognized primarily by RIG-I (10). RIG-I comprises two N-terminal caspase domains (CARDs), a helicase domain, and a C-terminal regulatory domain (CTD). The helicase and CTD domains of RIG-I recognize viral RNAs, exposing the two N-terminal CARDs, which interact with TRIM25, an E3-ubiquitin ligase (11, 12). TRIM25 deposits the K63-linked polyubiquitin chain on RIG-I CARDs, a phenomenon that induces RIG-I oligomerization and subsequent interaction with mitochondrial antiviral signaling (MAVS) protein (13, 14). As TRIM25 is one of the E3 ubiquitin ligases that play a crucial role in the RIG-I pathway, many viral proteins target TRIM25 to evade the RIG-I-mediated antiviral responses (15, 16). In particular, the V proteins of several members of the family *Paramyxoviridae* suppress RIG-I signaling by preventing TRIM25-mediated ligation of ubiquitin to RIG-I by forming complexes with both RIG-I and TRIM25 (17). Moreover, the NS1 protein of HRSV—a member of the family *Pneumoviridae*—interacts with TRIM25 and inhibits RIG-I ubiquitination (18). The ability to inhibit RIG-I signaling appears to be a common feature in the families *Paramyxoviridae* and *Pneumoviridae*. Therefore, we hypothesized that similar innate immune escape mechanisms might exist in HMPV as it is very similar to HRSV.

HMPV has a negative-sense single-stranded RNA genome, approximately 13 kb in length, comprising eight genes (3’-N-P-M -F-M2-SH-G-L-5’) that encode nine structural proteins (19), with M2 encoding two overlapping proteins, M2-1 and M2-2. However, unlike HRSV and members of the family *Paramyxoviridae*, HMPV does not encode non-structural proteins that are able to inhibit the innate immune cascade. Previous reports have suggested that some proteins, such as G, SH, and M2-2, modulate innate host immunity to favor HMPV infection *via* different mechanisms (5, 20). The attachment G protein, one of the two proteins responsible for viral entry, has been widely studied, as it plays a role in immune response evasion by inhibiting the IFN pathways. HMPV G protein can block RIG-I signaling by interacting with RIG-I (21, 22). Moreover, HMPV M2-2 protein interacts with MAVS protein and inhibits the production of MAVS-induced type I IFN, ultimately inhibiting the innate immune responses (23). However, the mechanisms underlying this HMPV-mediated immune inhibition have not been elucidated yet.

In this study, we sought to determine the mechanisms by which HMPV escapes the immune system. We identified an HMPV protein that inhibits the RIG-I/TRIM25 axis and elucidated the mechanism underlying the immune escape of HMPV. Notably, the mechanism was similar to that for previously reported V proteins of the family *Paramyxoviridae* and was also shared by the NS1 protein of HRSV. Our data provide mechanistic insights that would aid the development of therapeutic agents against infections attributed to members of the families *Paramyxoviridae* and *Pneumoviridae*.

## Materials and methods

### Cells and viruses

HEK293T, VeroE6/TMPRSS2 (JCRB, Osaka, Japan), and HEp-2 cells were cultured in Dulbecco’s modified Eagle’s medium supplemented with 10% heat-inactivated fetal bovine serum (FBS). The IRF and NF-κB-dual reporter A549 (A549-dual) cells (*In vivo*Gen, San Diego, CA, USA) were used to monitor the activities of the IRF and NF-κB pathways. rHPMV-GFP derived from the HMPV strain Jpn03-1 (GenBank accession number AB503857) has been previously described (24). Wild-type rHMPV-GFP (WT) and rHMPV-GFPΔM2-2 (ΔM2-2), in which M2-2 had been silenced (25), were propagated in VeroE6/TMPRSS2 cells. HRSV A2 strain was propagated in HEp-2 cells.

### Plasmids

To express cellular or viral proteins with or without FLAG, V5, HA, or Myc tags, mammalian expression plasmids were created by inserting a cDNA fragment carrying the respective gene into the multicloning site downstream of the cytomegalovirus enhancer chicken β-actin hybrid promoter of pCA7. cDNAs encoding human RIG-I and TRIM25 were purchased from OriGene (Rockville, MD, USA). Further, cDNA fragments encoding the RIG-I ORF [1–925 amino acid (aa)], RIG-I CARD [1–283 aa], TRIM25 [1–630 aa], and TRIM25 truncation mutants were synthesized using polymerase chain reaction (PCR). The cDNA encoding MAVS (derived from HeLa cells), NS1, and NS2 (derived from HRSV strain A2) were synthesized *via* reverse transcription (RT)-PCR. cDNA fragments encoding HMPV M2-2 deletion mutants were synthesized using PCR. pCA7, encoding P, M, F, M2-1, M2-2, SH, G, or L (derived from HMPV strain Jpn03-1) with or without the FLAG tag, were synthesized as described previously (25).

### Luciferase reporter assay

HEK293T cells (∼1 × 10^5^) were seeded in a 24-well plate and transfected with the following plasmids in triplicate: IFN-β promoter-driven firefly luciferase (Fluc) reporter [90 ng/well] (26, 27) or NF-κB-dependent Fluc reporter (TaKaRa Bio, Shiga, Japan) [90 ng/well] plasmid, Renilla luciferase (Rluc) pRL-TK control vector (Promega, Madison, WI, USA) [10 ng/well], plasmids encoding RIG-I signaling molecules (RIG-I CARD [10 ng/well] and/or TRIM25 [10 ng/well]) or MAVS protein (10 ng/well), and the plasmids encoding the viral proteins (NiV-V, MeV-V, HRSV-NS1, NS2, HMPV-N, P, M, F, M2-1, M2-2, SH, G, L, or M2-2 deletion mutants [280 ng/well]), using polyethyleneimine hydrochloride (MW 40,000) (PEI MAX) (#24765; Polysciences Inc., Warrington, PA, UK). An equal amount of DNA was used for transfection by adjusting the amount of the pCA7 empty plasmid. Cells were lysed 24 h post-transfection, and relative luciferase (Luc) activity was determined using the dual-Luc reporter assay system (Promega, Madison, WI, USA).

### Activities of IRF and NF-κB pathways

A549-dual cells were stably transfected with two inducible reporter constructs, and activation of the IRF and NF-κB pathways was monitored by evaluating luciferase (Luc) and secreted embryonic alkaline phosphatase (SEAP) activities, respectively, according to the manufacturer’s instructions (*In vivo*Gen).

### Immunoprecipitation

HEK293T cells (∼5.0 × 10^5^) seeded in 6-well plates were transfected with various combinations of plasmids (2 mg/well each) using PEI MAX. After incubation for an appropriate duration, the cells were lysed in 400 μL lysis buffer (50 mM Tris-HCl [pH 7.4], 150 mM NaCl, and 1% Triton X-100) supplemented with a protease inhibitor cocktail (Nacalai Tesque Inc., Kyoto, Japan). Cell lysates were then incubated with anti-FLAG, anti-V5, anti-Myc, or anti-HA mouse monoclonal antibody (MAb)-coated magnetic beads (MBL, Nagoya, Japan) at 4°C for 2 h. Beads were washed five times with lysis buffer and denatured in Laemmli sample buffer (50 mM Tris-HCl [pH 6.8], 2% sodium dodecyl sulfate (SDS), 0.1% bromophenol blue, 10% glycerol, and 5% 2-mercaptoethanol). The eluted proteins were subjected to immunoblot (IB) analysis. A portion of the cell lysate was also subjected to IB analysis.

### IB analysis

Samples were separated by SDS-polyacrylamide (10-16.5%) gel electrophoresis and electroblotted onto a membrane filter (Immobilon-P; MilliporeSigma, Burlington, MA, USA). Membranes were blocked with Blocking One (Nacalai Tesque) for 30 min, followed by incubation at 20–25°C with rabbit polyclonal antibodies against FLAG (MBL), V5 (MBL), Myc (MBL), HA (MBL), MAVS (D5A9E) (#24930, Cell Signaling Technology, Danvers, MA, USA), TRIM25 (#13773, Cell Signaling Technology) or a mouse monoclonal antibody against RIG-I (D-12) (sc-376845, Santa Cruz Biotechnology, Inc., Dallas, TX, USA) for 1 h. Membranes were then incubated at 20–25°C for 30 min with horseradish peroxidase-conjugated anti-mouse or anti-rabbit IgG antibodies (GE Healthcare, Chicago, IL, USA). Protein bands were visualized using enhanced chemiluminescence Western Lightning Ultra Substrate (PerkinElmer, Waltham, MA, USA) and FUSION-Solo S Imaging System (Vilber Lourmat Sté, Collégien, France).

### Statistical analysis

Data are presented as mean ± standard deviation (SD). Differences between two groups were analyzed using the Student’s *t*-test, whereas those between three or more groups were evaluated using Tukey’s test or Dunnett’s test. A *p* < 0.05 was considered statistically significant. All statistical analyses were performed using Microsoft Excel 2019 for Windows, version 10.

## Results

### HMPV M2-2 inhibits TRIM25-mediated RIG-I signaling preventing the activation of the IFN-β promoter

**Figure 1:**
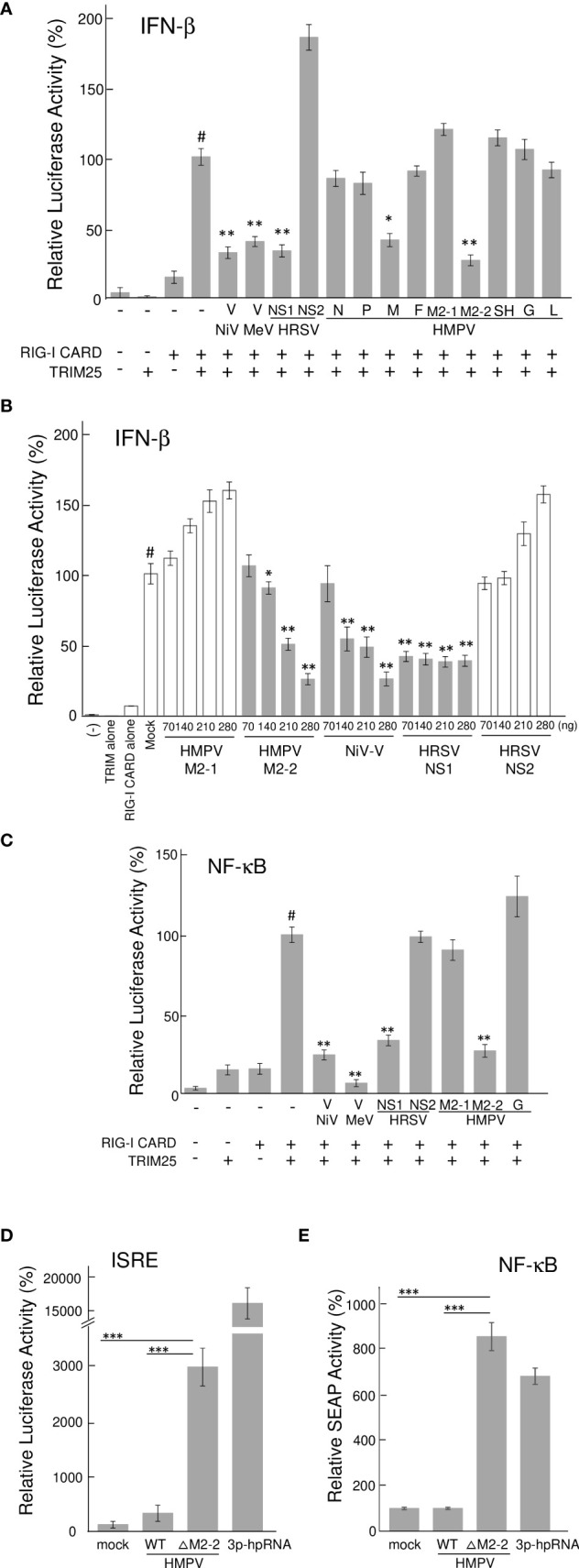
Effect of HMPV proteins on RIG–I CARD/TRIM25 signaling. **(A–C)** An IFN–β promoter–driven **(A, B)** or NF–κB–dependent **(C)** Fluc reporter plasmids were transfected into HEK293T cells with the internal control, pRL–TK, and indicated plasmids as described in the Reporter Assay section of the Materials and Methods. Fluc and Rluc activities were evaluated 24 h post–transfection. Relative luciferase activity was calculated as the ratio of Fluc activity to Rluc activity, *p < 0.05, **p < 0.01 *vs*. transfection with an empty vector instead of a plasmid encoding viral protein (#), Dunnett’s test. **(D, E)** A549–dual cells were infected with wild–type rHMPV–GFP (WT) or rHMPV–GFPΔM2–2 (ΔM2–2) at a multiplicity of infection (MOI) of 1 or transfected with 5 triphosphate hairpin RNA (3p–hpRNA) (100 ng/mL) as a positive control, and culture media were collected 36 h after infection or treatment. Activation of the IRF and NF–κB pathways in the culture media was evaluated by measuring the luciferase and SEAP activities, respectively, ***p < 0.001, Tukey’s test. Data are presented as mean ± SD of three independent experiments. HMPV, Human metapneumovirus, IFN–β, interferon–β, NF–κB, Nuclear factor–kappa B, IRF, interferon–regulatory factor, pRL–TK, Renilla luciferase plasmid, NiV, Nipah virus, MeV, measles virus. ISRE, interferon–stimulated response element.

To identify the HMPV proteins responsible for inhibiting RIG-I/TRIM25 signaling, we screened HMPV ORFs for their ability to block RIG-I/TRIM25 signaling reconstituted in HEK293T cells. In this reconstitution system, TRIM25 and RIG-I CARD were co-transfected into HEK293T cells along with an IFN-β promoter-driven firefly luciferase (Fluc) reporter plasmid and internal control (pRL-TK), in accordance with previously described procedures (17, 28). The effect of HMPV proteins on RIG-I CARD/TRIM25-mediated signaling was compared to that of Nipah virus (NiV) V, measles virus (MeV) V, and HRSV NS1 and NS2 proteins, which are well-known viral antagonists of RIG-I/TRIM25 signaling (17, 18, 29). As shown in **Figure 1A**, co-transfection with RIG-I CARD and TRIM25 resulted in a striking increase in IFN-β promoter activity (compared to transfection with RIG-I CARD or TRIM25 alone). This increase was suppressed upon co-transfection with M2-2 but not upon co-transfection with G, which is reportedly suppressed in other strains (21, 22). The most potent suppression was by M2-2, which exhibited dose-dependent inhibition, similar to that by NiV V (**Figure 1B**). HRSV NS1 exerted maximum inhibition at the lowest dose tested. A similar experiment was performed using the NF-κB-Fluc reporter plasmid. HMPV M2-2 suppressed RIG-I CARD/TRIM25-mediated NF-κB activation, similar to NiV V, MeV V, and HRSV NS1, whereas the HMPV-G protein did not (**Figure 1C**). To ascertain the effect of the M2-2 on the IRF and NF-kB pathways in infected A549 cells, luciferase and SEAP activities were evaluated in A549-dual cells infected with rHMPV-GFPΔM2-2 (ΔM2-2), in which the M2-2 ORF was silenced (24, 25). rHMPV–GFPΔM2–2 increased the luciferase and SEAP activities in cells by 9.3– and 8.4–fold, respectively (compared to wild–type rHMPV–GFP (WT)) (**Figures 1D, E**). 3p–hpRNA is a well–known activator of the RIG–I pathway. These results indicated that M2–2 inhibits RIG–I/TRIM25 signaling and suppresses the activation of the IFN–β and NF–κB pathways.

### M2–2 inhibits TRIM25–mediated RIG–I ubiquitination and the downstream RIG–I–MAVS protein interaction

**Figure 2:**
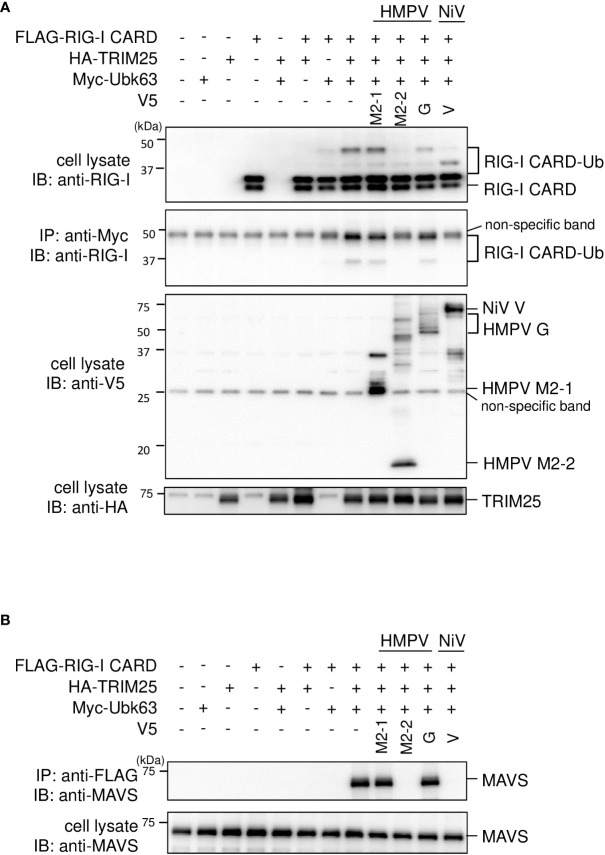
Effect of the HMPV M2–2 protein on RIG–I ubiquitination and subsequent RIG–I–MAVS protein interaction. **(A, B)** HEK293T cells were transfected with indicated plasmids. Cells were lysed using lysis buffer 24 h post–transfection. Cell lysates were subjected to immunoblot (IB) analysis with anti–RIG–I, anti–V5, or anti–HA antibodies, and immunoprecipitation (IP) analysis with anti–Myc, followed by IB with an anti–RIG–I antibody **(A)**. Cell lysates were subjected to IP with anti–FLAG, followed by IB with anti–MAVS protein antibody. A portion of the cell lysates was also subjected to IB **(B)**. HMPV, Human metapneumovirus, NiV, Nipah virus, MeV, measles virus.

We investigated the role of HMPV M2–2 in TRIM25–mediated K63–linked polyubiquitination of RIG–I and the downstream RIG–I–MAVS protein interaction. FLAG–tagged RIG–I CARD (FLAG–RIG–I CARD) and HA–tagged TRIM25 (HA–TRIM25) were co–transfected into HEK293T cells along with Myc–tagged K63–linked ubiquitin (Myc–Ubk63) and subjected to IB analysis with RIG–I antibodies. As previously reported (17, 28), ectopically expressed FLAG–RIG–I CARD was ubiquitinated, and further, the ubiquitination was enhanced by co–expressing Myc–Ubk63 or Myc–Ubk63 and HA–TRIM25 (**Figure 2A**). Thus, the minimal ubiquitination by RIG–I CARD alone was catalyzed by endogenous TRIM25. However, co–expression of HMPV M2–2 markedly suppressed the ubiquitination of FLAG–RIG–I CARD (this was similar to the results obtained upon NiV V co–expression). Conversely, HMPV G slightly suppressed FLAG–RIG-I CARD ubiquitination. Next, to detect the interaction between FLAG–RIG–I CARD and endogenous MAVS protein, cell lysates were subjected to IP with an anti–FLAG antibody, followed by IB analysis with an antibody against MAVS protein. Co–expression of HA–TRIM25 and Myc–Ubk63 resulted in induced RIG-I CARD polyubiquitination, indicating an interaction between FLAG–RIG–I CARD and the MAVS protein (**Figure 2B**). However, this interaction was disrupted upon co–transfection with HMPV M2-2 or NiV V. In contrast, HMPV M2-1 and HMPV G did not suppress the interaction between the RIG–I CARD and MAVS protein. These results were also in alignment with those presented in **Figure 2A**. Thus, we can infer that M2–2 interfered with the RIG–I CARD–MAVS protein interaction, possibly by regulating the ubiquitination status of RIG–I CARD.

### TRIM25 is a potential target of M2–2 protein

**Figure 3:**
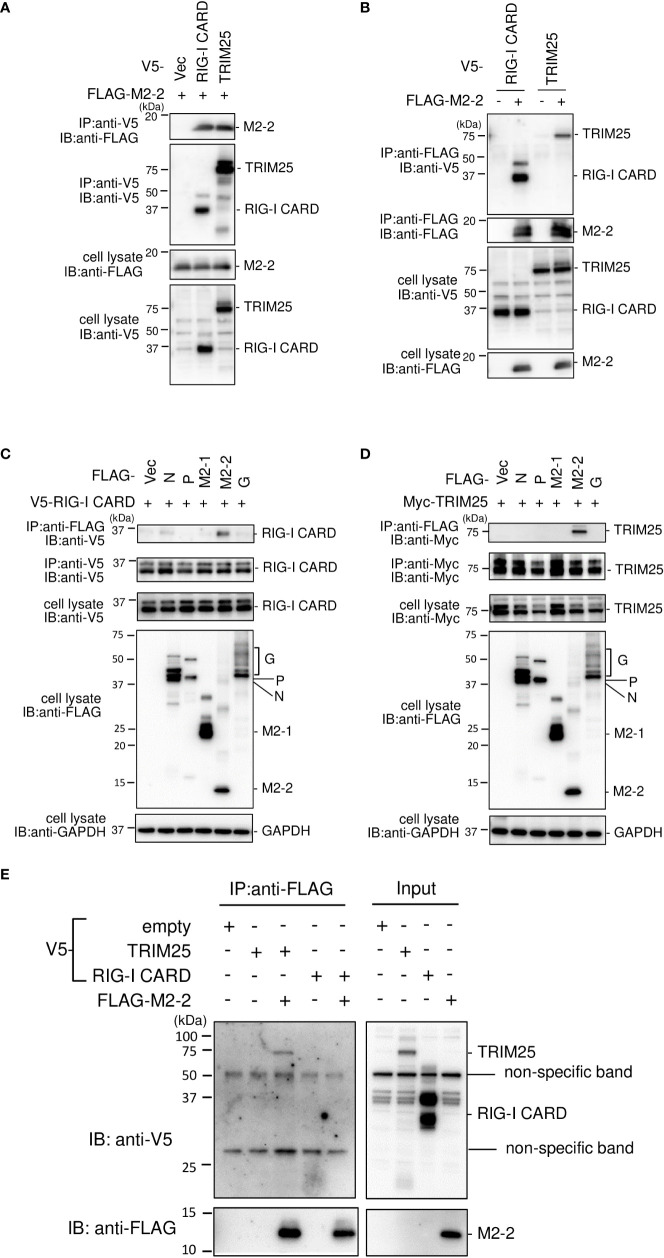
Interactions among the HMPV M2–2, RIG–I, and TRIM25 proteins. **(A–D)** HEK293T cells were transfected with the indicated plasmids. Cells were lysed in lysis buffer 24 h post–transfection and subjected to IP with anti–V5 **(A)**, anti–FLAG **(B)**, anti–FLAG, anti–V5 **(C)**, anti–FLAG, or anti–Myc antibodies **(D)**, followed by immunoblotting (IB) with anti–FLAG, anti–V5, or anti–Myc antibodies. **(E)** V5–TRIM25, V5–RIG–I CARD, and FLAG–M2–2 were synthesized using a wheat germ cell–free expression system. Then, the *in vitro* transcription/translation products were mixed in various combinations and subjected to immunoprecipitation (IP) analysis with an anti–FLAG antibody, followed by IB with an anti–V5 antibody. A portion of the cell lysates **(A–D)** or the *in vitro* transcription/translation products (Input) **(E)** was also subjected to IB.

To investigate the interactions among M2–2, RIG–I CARD, TRIM25, FLAG–tagged M2–2 (FLAG–M2–2) was transfected into HEK293T cells with V5–tagged RIG–I CARD (V5–RIG–I CARD) or V5–tagged TRIM25 (V5–TRIM25). Transfected cells were subjected to IP. As shown in **Figure 3A**, FLAG–M2–2 co–immunoprecipitated with anti–V5 antibodies in cells transfected with either V5–RIG–I CARD or V5–TRIM25. Conversely, both V5–RIG–I CARD and V5–TRIM25 co–immunoprecipitated with anti–FLAG antibodies (**Figure 3B**). Further IP experiments on cells transfected with V5–RIG–I CARD or Myc–tagged TRIM25 (Myc–TRIM25) and various FLAG–tagged HMPV proteins showed that only M2–2 protein interacted with both RIG–I CARD and TRIM25 (**Figures 3C, D**), suggesting that M2–2 specifically interacts with RIG–I CARD and TRIM25. To determine whether these interactions were direct, similar experiments were performed for mixtures of either FLAG–M2–2 and V5–RIG–I CARD or FLAG–M2–2 and V5–TRIM25, which were synthesized using a wheat germ cell–free expression system. **Figure 3E** shows, only V5–TRIM25 was immunoprecipitated upon using an anti–FLAG antibody, indicating that M2–2 interacted directly with TRIM25 but not RIG–I CARD.

### M2–2 binds to the SPRY domain of TRIM25

**Figure 4:**
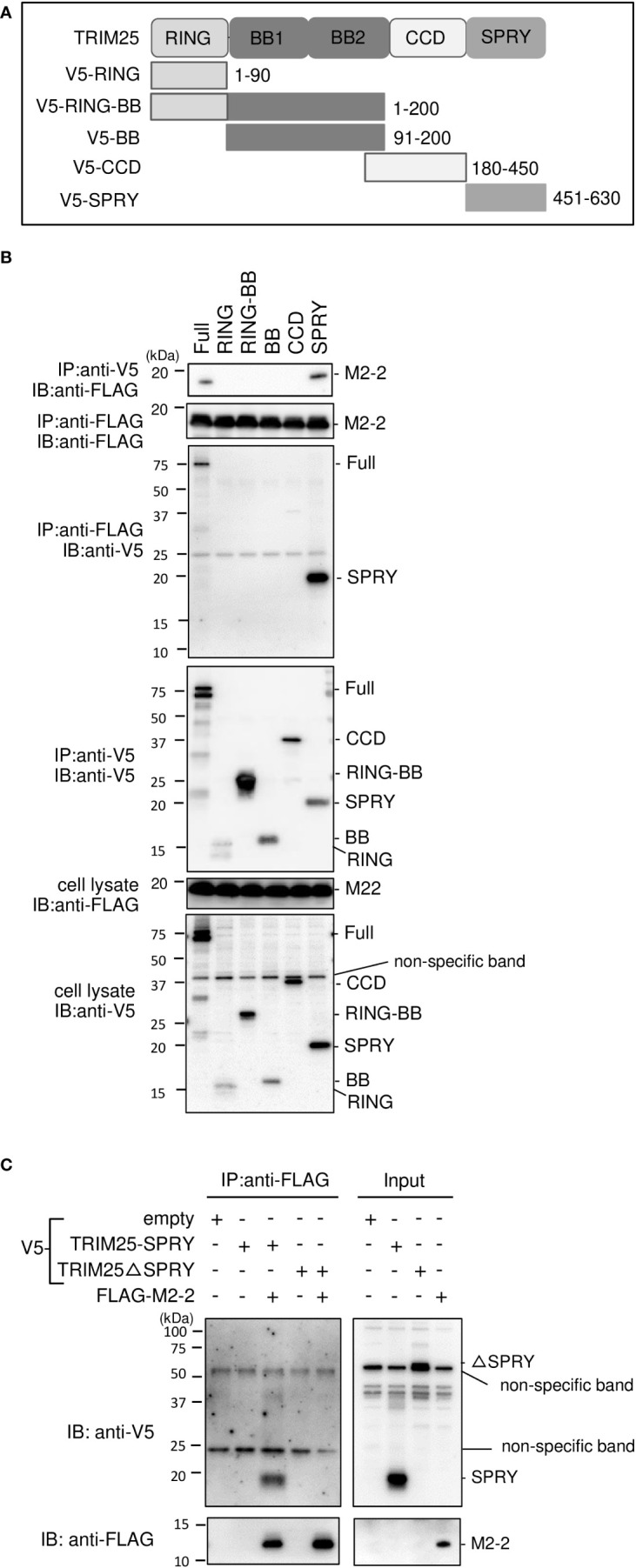
Interactions between HMPV M2–2 protein and TRIM25 truncation mutants. **(A)** Schematic diagram of V5–tagged truncation mutants of TRIM25. **(B)** HEK293T cells were transfected with the indicated plasmids and lysed 24 h post–transfection. Cell lysates were subjected to IP with anti–V5 or anti–FLAG antibodies, followed by immunoblotting (IB) with an anti–V5 antibody. **(C)** V5–TRIM25, V5–RIG–I CARD, and FLAG–M2–2 were synthesized using a wheat germ cell–free expression system. Then, the *in vitro* transcription/translation products were mixed in various combinations and subjected to immunoprecipitation (IP) analysis with an anti–FLAG antibody, followed by IB with an anti–V5 antibody. A portion of the total cell lysates **(B)** or the *in vitro* transcription/translation products (Input) **(C)** was also subjected to IB.

TRIM25 comprises five domains, i.e., RING (RING–finger domain), BB (B box) 1, BB (B box) 2, CCD (coiled–coil domain), and SPRY (SPRY) (30) (**Figure 4A**). To identify the domains that interact with M2–2, we created TRIM25 deletion mutants and performed IP experiments. V5–tagged TRIM25 deletion mutants, RING and BB1, were transfected into HEK293T cells, together with FLAG–M2–2. FLAG–M2–2 was immunoprecipitated upon using the anti–V5 antibody in cells transfected with FLAG–SPRY but not with RING, BB1, BB2, or CCD, suggesting that the SPRY domain of TRIM25 interacts with M2–2 (**Figure 4B**). Furthermore, to determine whether this interaction was direct, similar experiments were performed for mixtures of either FLAG–M2–2 and V5–TRIM25 SPRY or FLAG–M2–2 and V5–TRIM25ΔSPRY (lacking SPRY), which were synthesized using a wheat germ cell–free expression system (**Figure 4C**). Expectedly, V5–SPRY was immunoprecipitated upon using the anti–FLAG antibody, whereas V5–TRIM25ΔSPRY was not, indicating that the interaction between M2–2 and TRIM25 SPRY is direct.

### M2–2 inhibits MAVS–mediated IFN–β activation

**Figure 5:**
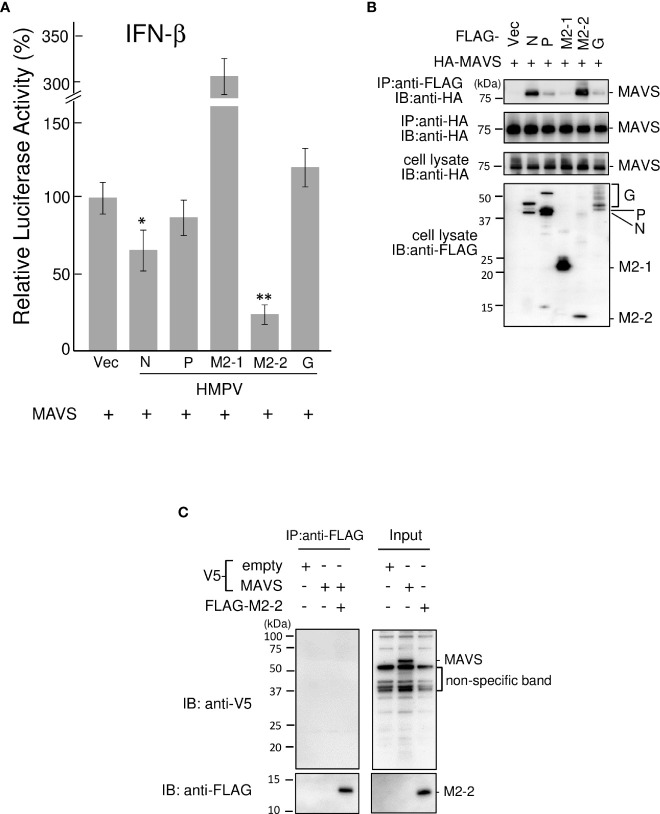
Effect of the HMPV M2–2 protein on MAVS–mediated signaling. **(A)** An IFN–β promoter–driven Fluc reporter plasmid was transfected into HEK293T cells with the internal control, pRL–TK, and the indicated plasmids. Fluc and Rluc activities were evaluated 24 h post–transfection. Relative luciferase activity was calculated as the ratio of Fluc activity to Rluc activity, *p < 0.05, **p < 0.01 *vs*. transfection with empty vector, Dunnett’s test. **(B)** HEK293T cells were transfected with the indicated plasmids. Cells were lysed in lysis buffer 24 h post–transfection and subjected to immunoprecipitation (IP) with anti–FLAG or anti–HA antibodies (**A**), followed by immunoblotting (IB) with an anti–HA antibody. **(C)** V5–MAVS and FLAG–M2–2 were synthesized using a wheat germ cell–free expression system. Then, the *in vitro* transcription/translation products were mixed in various combinations and subjected to IP with an anti–FLAG antibody, followed by IB with anti–V5 or anti–FLAG antibodies. A portion of cell lysates **(A, B)** or the *in vitro* transcription/translation products (Input) **(C)** was also subjected to IB. HMPV, Human metapneumovirus, MAVS, mitochondrial antiviral signaling proteins.

Ren et al. reported that the M2–2 protein of the HMPV strain CAN97–83 suppresses MAVS—which acts downstream of the RIG–I pathway—by targeting the MAVS protein (23) and that M2–2 suppresses RIG–I–mediated signaling. Thus, we tested the ability of the M2–2 protein of the JPN03–1 HMPV strain to block MAVS in HEK293T cells. Transfection with a plasmid expressing MAVS protein alone markedly enhanced the IFN–β promoter activity even in the absence of transfection of the upstream components (**Figure 5A**). This activation was suppressed upon co–transfection with N or M2–2 proteins. IP experiments on cells transfected with HA–tagged MAVS (HA–MAVS) and various FLAG–tagged HMPV proteins showed that N and M2–2 proteins interact with MAVS proteins (**Figure 5B**), suggesting the specificity of the interactions between M2–2 and MAVS proteins. Furthermore, to determine whether these interactions are direct, similar experiments were performed for mixtures of FLAG–M2–2 and V5–MAVS, which were synthesized using a wheat germ cell–free expression system (**Figure 5C**). V5–MAVS was not immunoprecipitated upon using the anti–FLAG antibody, indicating that the interactions between M2–2 and MAVS proteins were indirect. These results suggest that the M2–2 protein of the JPN03–1 strain also inhibits MAVS by indirectly interacting with MAVS proteins.

### M2–2 functional domains inhibit RIG–I CARD/TRIM25–mediated signaling

**Figure 6:**
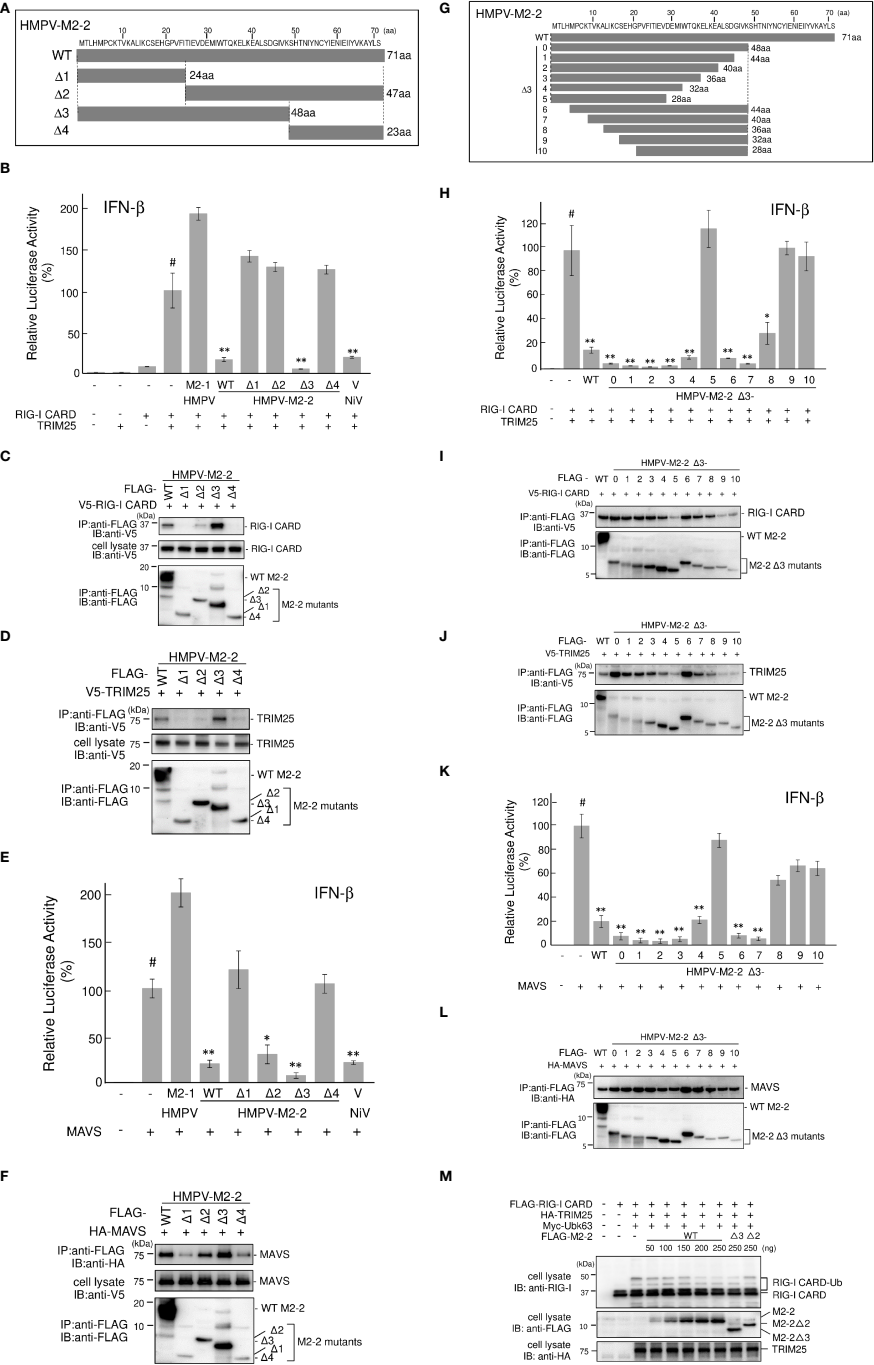
HMPV M2–2 domains that inhibit RIG–I CARD/TRIM25–mediated IFN–β activation. **(A, G)** Schematic diagram of FLAG–tagged deletion mutants of M2–2. **(B, E, H, K)** An IFN–β promoter–driven Fluc reporter plasmid was transfected into HEK293T cells with the internal control, pRL–TK, and the indicated plasmids. Fluc and Rluc activities were evaluated 24 h post–transfection. Relative luciferase activity was calculated as the ratio of Fluc activity to Rluc activity, *p < 0.05, **p < 0.01 *vs*. transfection with empty vector (#). Dunnett’s test. Data are presented as the mean ± SD of three independent experiments. **(C, D, F, I, J, L)** HEK293T cells were transfected with the indicated plasmids and lysed 24 h post–transfection. Cell lysates were subjected to immunoprecipitation (IP) with an anti–FLAG antibody, followed by immunoblotting (IB) with anti–V5, anti–HA, or anti–FLAG antibodies. A portion of the cell lysates was also subjected to IB. (**M**) HEK293T cells were transfected with the indicated plasmids and lysed 24 h post–transfection. Cell lysates were subjected to IB with anti–RIG–I, anti–FLAG, or anti–HA antibodies. HMPV, Human metapneumovirus, IFN–β, interferon–β, MAVS, mitochondrial antiviral signaling proteins.

To examine whether the ability of M2–2 to inhibit MAVS is important for the inhibition of RIG–I CARD/TRIM25 signaling, we determined the functional domain(s) of M2–2 that inhibit RIG–I CARD/TRIM25 and MAVS–mediated signaling. Thus, we created four M2–2 deletion mutants (Δ1–4, **Figure 6A**) and examined their inhibitory effects on RIG–I CARD/TRIM25– and MAVS–induced IFN–β promoter activation compared to WT, M2–1, and NiV V, which was previously reported to inhibit MAVS (31). As shown in **Figure 6B**, Δ3 exhibited an inhibitory effect on the RIG–I CARD/TRIM25–induced IFN–β promoter activity, similar to that induced by WT M2–2 and NiV, whereas Δ1, Δ2, and Δ4 did not. Additionally, we evaluated their interactions with RIG–I CARD, TRIM25, and MAVS. IP experiments showed that Δ3 strongly binds to RIG–I CARD and TRIM25, whereas Δ1, Δ2, and Δ4 exhibited only a small or negligible ability to bind to RIG–I CARD and TRIM25 (**Figures 6C, D**), which is consistent with the results shown in **Figure 6B**. This suggests that the 1–48 aa region is critical for inhibiting RIG–I CARD/TRIM25 signaling. In contrast—in a manner similar to WT M2–2 and NiV V—Δ2 inhibited MAVS–induced IFN–β promoter activity (**Figure 6E**). IP experiments showed that Δ2 binds to MAVS proteins (**Figure 6F**). However, it should be noted that Δ2, which retained its inhibitory effect on MAVS and the ability to bind to MAVS proteins, did not exhibit an inhibitory effect on RIG–I CARD/TRIM25 signaling and the ability to bind to RIG–I CARD and TRIM25. Collectively, these results suggest that the inhibition of RIG–I CARD/TRIM25–mediated signaling is possibly independent of the ability of M2–2 to inhibit MAVS.

To confirm this, we performed similar experiments using finer Δ3 deletion mutants (**Figure 6G**). Similar to WT M2–2, Δ3–1, Δ3–2, Δ3–3, Δ3–4, Δ3–6, Δ3–7, and Δ3–8 exhibited an inhibitory effect on RIG–I CARD/TRIM25–induced IFN–β promoter activity and ability to bind to RIG–I CARD and TRIM25, whereas Δ3–5, Δ3–9, and Δ3–10 did not, suggesting that the 13–32 aa region is important for the inhibition of RIG–I CARD/TRIM25 signaling (**Figures 6H**–**J**). Although Δ3–8 retained the ability to bind to the MAVS protein, it did not inhibit MAVS (**Figures 6K, L**). Notably, Δ3–8, which did not retain its inhibitory effect on MAVS, exhibited an inhibitory effect on RIG-I CARD/TRIM25 signaling. Collectively, these results suggest that the inhibition of RIG–I CARD/TRIM25 signaling by M2–2 is not due to MAVS inhibition. Furthermore, TRIM25–mediated RIG–I CARD ubiquitination was substantially and dose–dependently suppressed in response to increased M2–2 protein expression (**Figure 6M**). Ubiquitination was also suppressed in Δ3–transfected cells as efficiently as in WT M2–2–transfected cells, whereas it was not suppressed in cells transfected with Δ2. Therefore, these results suggest that the inhibition of RIG–I CARD/TRIM25 signaling by M2–2 is not due to MAVS inhibition.

### HMPV M2–2 is incorporated into the RIG–I CARD/TRIM25 complex

**Figure 7:**
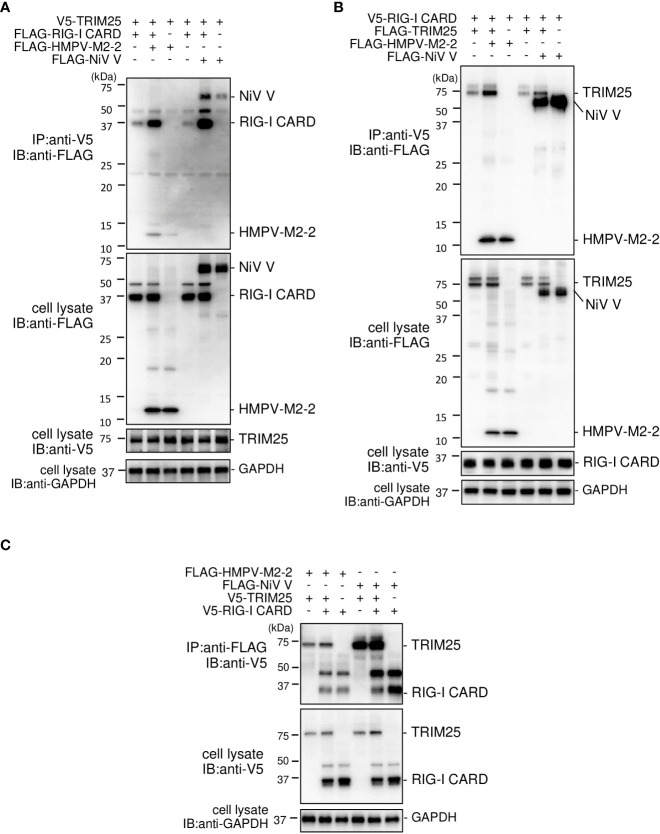
Interaction between the HMPV M2–2 protein and the RIG–I CARD/TRIM25 complex. **(A–C)** HEK293T cells were transfected with the indicated plasmids. Cells were lysed in lysis buffer 24 h post–transfection and subjected to immunoprecipitation (IP) with anti–V5 **(A, B)** or anti–FLAG **(C)**, followed by immunoblotting (IB) with anti–FLAG or anti–V5 antibodies. A portion of the cell lysates was also subjected to IB. HMPV, Human metapneumovirus, IFN–β, interferon–β, NiV, Nipah virus.

The SPRY domain of TRIM25 is necessary for TRIM25–RIG–I interactions (30). Therefore, the binding between M2–2 and SPRY may affect the interaction between RIG–I and TRIM25. To evaluate this possibility, FLAG–M2–2 was transfected into HEK293T cells with various plasmid combinations, including either V5–TRIM25 and FLAG–RIG–I CARD or V5–RIG–I CARD and FLAG–TRIM25, and the transfected cells were subjected to IP analysis. FLAG–RIG–I CARD was immunoprecipitated along with V5–TRIM25 (**Figure 7A**). The amount of co–immunoprecipitated FLAG–RIG–I CARD was increased upon co–transfection with FLAG–M2–2. In addition, FLAG–TRIM25 was immunoprecipitated along with V5–RIG–I CARD (**Figure 7B**), and the amount of co–immunoprecipitated FLAG–TRIM25 also increased upon co–transfection with FLAG–M2–2. Conversely, both V5–RIG–I CARD and V5–TRIM25 were immunoprecipitated along with FLAG–M2–2 when FLAG–M2–2 was transfected into HEK293T cells together with V5–RIG–I CARD and V5–TRIM25 **Figure 7C**. These results were similar to those obtained for NiV V–transfected cells, suggesting that M2–2 is incorporated inside the RIG–I CARD/TRIM25 complex and may stabilize the complex.

### HRSV NS1 is incorporated into the RIG-I CARD/TRIM25 complex

**Figure 8:**
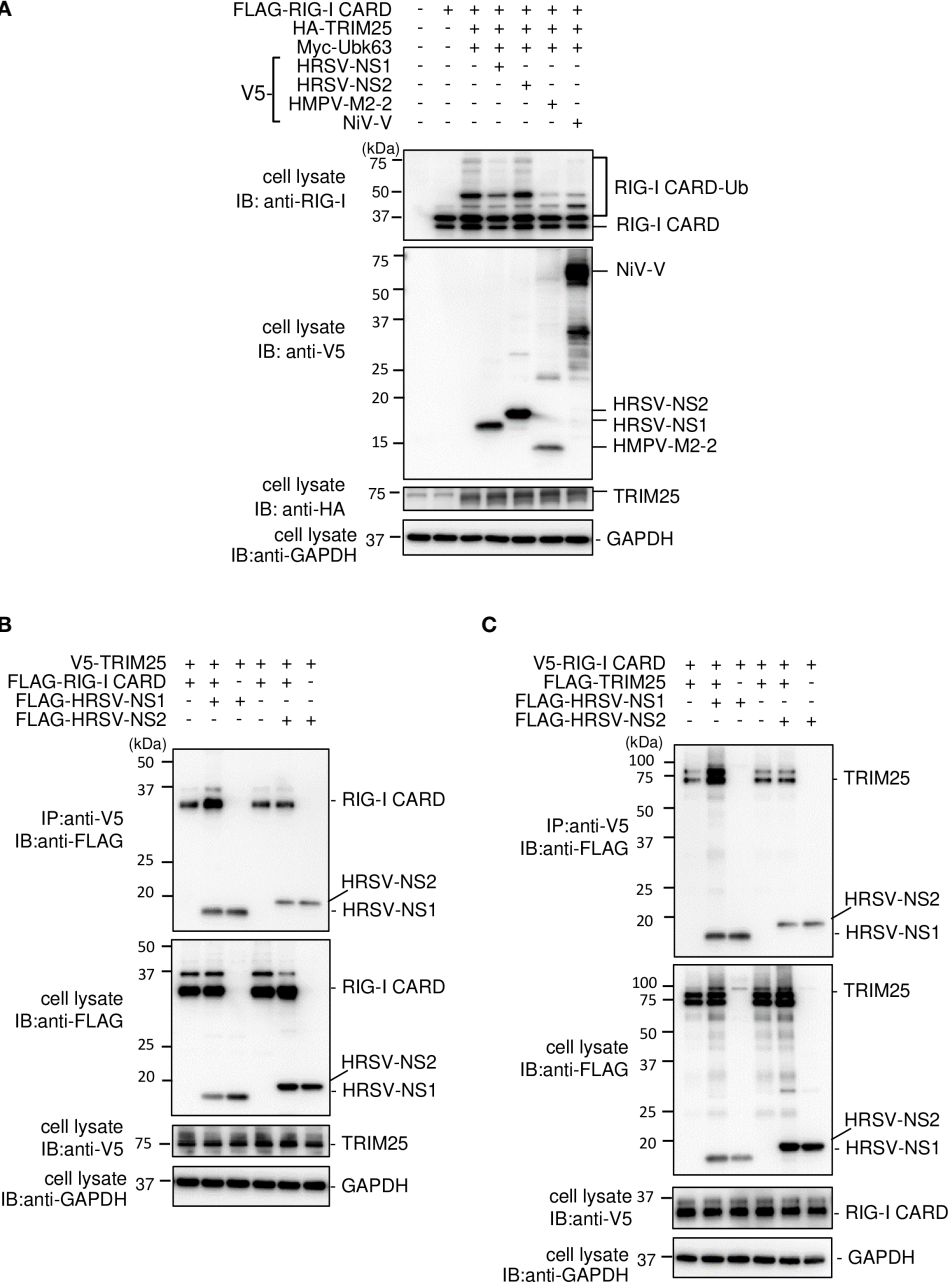
Interaction between the HRSV NS1 protein and the RIG–I CARD/TRIM25 complex. **(A)** HEK293T cells were transfected with the indicated plasmids. Cells were lysed in lysis buffer 24 h post–transfection and subjected to immunoblotting (IB) analysis with anti–RIG–I, anti–V5, or anti–HA antibodies. **(B, C)** HEK293T cells were transfected with the indicated plasmids. Cells were lysed in lysis buffer 24 h post–transfection and subjected to immunoprecipitation (IP) with an anti–V5 antibody, followed by IB with an anti–FLAG antibody. A portion of the cell lysates was also subjected to IB. HRSV, human respiratory syncytial virus.

HRSV NS1 protein has been reported to target TRIM25 to inhibit RIG–I ubiquitination and the subsequent RIG–I–mediated antiviral signaling (18). To confirm this, we performed IB analysis using the same procedure as in **Figure 2A**. HRSV NS1 suppressed the ubiquitination of FLAG–RIG–I CARD—similar to the results obtained upon the HMPV M2–2 or NiV V co–expression, whereas HRSV NS2 did not (**Figure 8A**). This result suggests that NS1 may also affect RIG–I/TRIM25–mediated signaling *via* a mechanism similar to that employed by HMPV M2–2 and NiV V. Next, to examine the effect of NS1 on the interaction between RIG–I and TRIM25, FLAG–HRSV NS1 or –HRSV NS2 was transfected into HEK293T cells with either V5–TRIM25 and FLAG–RIG–I CARD or V5–RIG–I CARD and FLAG–TRIM25. Transfected cells were subjected to IP with an anti–V5 antibody. FLAG–RIG–I CARD was immunoprecipitated along with V5–TRIM25 (**Figure 8B**). The amount of immunoprecipitated FLAG– RIG–I CARD increased upon co–transfection with FLAG–NS1. In addition, FLAG–TRIM25 was immunoprecipitated along with V5–RIG–I CARD, and the amount of co–immunoprecipitated FLAG– RIG–I CARD was also increased upon co–transfection with NS1 (**Figure 8C**). Co–immunoprecipitated FLAG–HRSV–NS2 was detected in cells co–transfected with V5–RIG–I CARD and V5–TRIM25, however, the amount of precipitated proteins was small. Additionally, NS2 did not affect the RIG–I CARD/TRIM25 complex formation. This result is consistent with those in **Figure 1A**, where NS2 did not inhibit RIG–I CARD/TRIM25–induced IFN–β promoter activity, which has previously been reported to be suppressed in other strains (29). These results suggest that NS1, but not NS2, is also incorporated into the RIG–I CARD/TRIM25 complex and that it may stabilize this complex.

## Discussion

Innate immunity functions as the first line of host defense against invading pathogens, as well as a critical component in regulating adaptive immunity. The effectiveness of the innate immune response against viral infection depends on the interactive nature of virus components with the host innate antiviral immune systems, including the type I IFN production system (32). Although HMPV induces poor innate immune responses (5), the role of individual HMPV proteins in modulating host innate immune responses remains largely unclear. RIG–I establishes an antiviral state by triggering signaling cascades that induce the expression of type I IFNs and inflammatory cytokines (6–9). The ability to inhibit RIG–I signaling is seemingly a common feature in the families *Paramyxoviridae* and *Pneumoviridae* in the order *Mononegavirales*. Therefore, we hypothesized that similar innate immune escape mechanisms are at play in HMPV as it is remarkably similar to HRSV and parainfluenza viruses, members of the *Pneumoviridae* and *Paramyxoviridae* families, respectively. In this study, we screened HMPV ORFs to identify HMPV proteins that inhibit the RIG–I/TRIM25 signaling axis and identified the M2–2 protein as a negative regulator of RIG–I/TRIM25 signaling. Compared to WT HMPV, recombinant ΔM2–2 HMPV, in which M2–2 expression was abrogated, activated transcription factors downstream of the RIG–I signaling pathway in A549 cells. In this study, we focused on the mechanism(s) underlying RIG–I inhibition by M2–2.

To reveal the molecular mechanism(s) by which the M2–2 protein inhibits RIG–I/TRIM25–mediated signaling leading to IFN–β activation, we first examined the effect of M2–2 on RIG–I activation and the downstream protein MAVS, according to a previously described procedure (17,28). M2–2 overexpression inhibited K63–linked polyubiquitination of RIG–I–CARD and CARD–dependent interactions with MAVS. Additionally, we assessed the interaction between M2–2 and RIG–I or TRIM25 by IP using extracts from cells transfected with M2–2 together with RIG–I CARD, TRIM25, or TRIM25 truncation mutants. These experiments showed that M2–2 interacts with RIG–I CARD and TRIM25 SPRY and that M2–2–TRIM25 SPRY interactions were direct, whereas M2–2–RIG–I CARD interactions were not. The TRIM25 SPRY domain is responsible for interactions with RIG–I CARD (30), therefore, the M2–2 protein may bind to the interface between RIG–I CARD and TRIM25. In this study, M2–2 enhanced the stabilization of the RIG–I CARD–TRIM25 complex. Therefore, HMPV M2–2 likely inhibits the K63–linked polyubiquitination of RIG–I by interacting with RIG–I and TRIM25 to form a complex.

This study has revealed that the mechanism underlying HMPV–M2–2 protein–mediated innate immune inhibition is similar to that by V proteins of several members of the family *Paramyxoviridae* (17). However, the previous study did not examine whether V directly binds to RIG–I and TRIM25. In addition, V proteins also reportedly inhibit MAVS–mediated signaling, leading to the production of type I IFNs (31), however, the impact of MAVS inhibition on RIG–I signaling inhibition has not been investigated. In this study, we characterized the detailed mechanisms underlying the inhibition of RIG–I signaling/MAVS by HMPV M2–2. Additionally, we also revealed that the HRSV NS1 protein also interacts with TRIM25, inhibiting RIG–I ubiquitination, which is consistent with the findings of a previous report (18). Furthermore, we revealed that the HRSV NS1 protein is incorporated into the RIG–I CARD/TRIM25 complex and may stabilize these complexes in a manner similar to the HMPV M2–2 protein.

In addition to viruses of the families *Paramyxoviridae* and *Pneumoviridae*, several RNA viruses have evolved strategies that favor their proliferation by targeting molecules such as RIG–I, TRIM25, and MAVS proteins. The NS1 protein of the influenza A virus (IAV), belonging to the *Orthomyxoviridae* family, interacts with TRIM25 and Riplet, repressing K63–linked ubiquitination of RIG–I (33, 34). NS1 also promotes TRIM25–mediated ubiquitination *via* strain–specific direct interaction with the second CARD of RIG–I (35). The SPRY domain is also a target of the severe acute respiratory syndrome (SARS) N protein, however, in contrast to that of the M2–2 protein, the interaction between SARS–N and TRIM25 prevents the association of TRIM25 with RIG–I (36). Despite these differences, the effects of IAV NS1, SARS N, paramyxovirus V, HRSV NS1, and HMPV M2-2 proteins are similar, i.e., inhibition of RIG–I signaling/MAVS by preventing TRIM25–mediated ubiquitination of RIG–I CARDs. Several disease–causing viruses in humans target TRIM25–mediated ubiquitination in the innate immune response. Therefore, targeting this inhibitory mechanism may aid the development of novel broad–spectrum antivirals. RIG–I may be a promising target because arenavirus Z protein and herpesvirus U11 are also pathogenic viral proteins that interact with RIG–I and suppress RIG–I signaling (37, 38). The mechanism by which the M2–2 protein inhibits the type I IFN signaling cascade, i.e., by antagonizing TLR7/9 signaling, has been reported (25, 37). However, our findings present a novel mechanism for M2–2–mediated inhibition of RIG–I signaling that is MAVS–independent and specific to the TRIM25–mediated regulatory step in the RIG–I activation pathway. HMPV may evade the innate immune system by leveraging both of these mechanisms.

Ren et al. showed that the M2–2 protein interacts with the MAVS protein, but not RIG–I, to inhibit RIG–I– and MAVS–mediated IFN–β promoter and NF–kB activity (23). We also confirmed an inhibitory effect on MAVS–induced IFN–β promoter activity and a significant, albeit indirect, interaction between M2–2 and the MAVS protein. Thus, we examined the role of M2–2–mediated MAVS inhibition in RIG–I CARD/TRIM25 signaling inhibition using M2–2 deletion mutants. Our results suggested that the ability of M2–2 to inhibit MAVS is not necessary for the negative regulation of the RIG–I CARD/TRIM25 axis. Additionally, previous studies have reported that the C–terminal half of HMPV M2–2 is responsible for the inhibitory effect on MAVS–induced activation of the IFN–β promoter (23). However, our data indicate that the N–terminal half of HMPV M2–2 is responsible for the inhibitory effect on RIG–I CARD/TRIM25 signaling and MAVS. We speculate that these results may be due to the different HMPV strains used in other studies. In comparison with the CN97/83 strain used in other studies, the Jpn03–1 strain used in our study is identical to the CAN97–83 strain in M2–1 ORF and the region of overlap between the M1 and M2 proteins but has a single amino acid difference at position 58 in M2–2 (I and L at this position in Jpn03–1 and CAN97–83, respectively), which is located in the C–terminal half of the M2–2 protein (71 aa). Thus, the reasons for these conflicting results remain unclear.

Nevertheless, the current study has limitations. M2–2 silencing induced the activation of transcription factors (IRF and NF–kB) downstream of RIG–I signaling in A549 cells. This activation may occur through two M2–2 activities, partly explaining why M2–2 deletion attenuates the virus (20). The first is through the inhibition of the RIG–I/TRIM25 signaling axis, in our study, M2–2 was the most potent inhibitor among all the proteins encoded by the HMPV genome in HEK293T cells. The second is through the inhibition of viral RNA synthesis. A previous study has shown that the ΔM2–2 virus produces higher levels of viral mRNAs in Vero cells than the WT virus (39). Additionally, the inhibitory effect of M2–2 on viral RNA synthesis was also confirmed by experiments with an HMPV minigenome construct carrying a luciferase reporter gene (40). In our study, A549 cells infected with the ΔM2–2 virus showed increased viral RNA synthesis. This may increase pathogen–associated molecular patterns (PAMP) levels, thereby inducing IFN–β activation. However, it is difficult to determine the exact contribution of each activity to the inhibition of IFN–β activation. Furthermore, knockout of the M2–2 gene results in the attenuation of virus pathogenicity. Therefore, the recombinant virus, whose M2–2 gene is silenced, is a candidate for attenuated virus vaccine. However, the M2–2–knockout recombinant HMPV show too poor growth in cell culture and cannot be prepared as a vaccine (5, 20, 23). The M2–2 protein is a multifunctional protein that exerts an anti–IFN effect, regulating viral RNA synthesis (39, 41). Such various functions collectively contribute to virus pathogenicity, resulting in the overattenuation of recombinant viruses. Thus moderately attenuated recombinant viruses, which could be created by silencing only a single function with other functions remaining, might be suitable for vaccine development. For this purpose, it would be necessary to determine domains or amino acid residues important for maintaining each function of the M2–2 protein.

In conclusion, we revealed that the HMPV M2–2 protein acts as an IFN antagonist that inhibits RIG–I CARD–dependent interactions with the MAVS protein by blocking TRIM25–mediated RIG–I ubiquitination, possibly by forming a stable complex with both RIG–I and TRIM25. Similarly, HRSV NS1 also formed a stable complex with RIG–I CARD/TRIM25 and inhibited RIG–I ubiquitination. Notably, the inhibitory actions of HMPV M2–2 and HRSV NS1 proteins are shared among several members of the *Paramyxoviridae* family. We identified that HMPV, similar to HRSV and parainfluenza viruses, has an innate immune escape mechanism similar to theirs. Several disease–causing human viruses target TRIM25 ubiquitination in the innate immune response, therefore, targeting this inhibitory mechanism may aid the development of novel broad–spectrum antivirals.

Furthermore, moderately attenuated recombinant viruses, which could be created by silencing only a single function while preserving other functions, might be suitable for vaccine development.

## Data availability statement

The datasets presented in this study can be found in online repositories. The names of the repository/repositories and accession number(s) can be found in the article.

## Author contributions

YT and TK designed the study and prepared and revised the manuscript. YT, NM, YK, BG, and TK analyzed the data. YT, NM, YK, and TK performed the experiments. All authors contributed to the article and approved the submitted version.

## Funding

This work was supported by JSPS KAKENHI Grant Number 20K17463.

## Acknowledgments

We thank the Division of Bioresearch in Life Science Laboratory (Fukui University) and the Division of Advanced Research Promotion (Aichi Medical University) for technical instructions and assistance.

## Conflict of interest

The authors declare that the research was conducted in the absence of any commercial or financial relationships that could be construed as a potential conflict of interest.

## Publisher’s note

All claims expressed in this article are solely those of the authors and do not necessarily represent those of their affiliated organizations, or those of the publisher, the editors and the reviewers. Any product that may be evaluated in this article, or claim that may be made by its manufacturer, is not guaranteed or endorsed by the publisher.
